# A comprehensive mobility discharge assessment framework for older adults transitioning from hospital-to-home in the community—What mobility factors are critical to include? Protocol for an international e-Delphi study

**DOI:** 10.1371/journal.pone.0267470

**Published:** 2022-09-22

**Authors:** Michael E. Kalu, Vanina Dal Bello-Haas, Meridith Griffin, Jenny Ploeg, Julie Richardson

**Affiliations:** 1 School of Rehabilitation Science, Faculty of Health Sciences, McMaster University, Hamilton, Ontario, Canada; 2 Department of Health, Aging & Society, Faculty of Social Science, McMaster University Hamilton, Ontario, Canada; 3 School of Nursing, Faculty of Health Sciences, McMaster University Hamilton, Ontario, Canada; 4 Department of Health Evaluation and Impact, Faculty of Health Sciences, McMaster University, Ontario, Canada; IRCCS E. Medea, ITALY

## Abstract

**Background:**

Mobility deficits have been identified as an independent risk factor for hospital readmission for adults ≥65 years. Despite evidence indicating how determinants additively influence and predict mobility, no hospital-to-home care transition models comprehensively assess all seven mobility determinants, cognitive, financial, environmental, personal, physical, psychological, and social. There is currently a lack of clarity regarding what factors clinicians and researchers should evaluate for each mobility determinant. The purpose of this e-Delphi study is to prioritize and reach consensus on the factors for each mobility determinant that are critical to assess as part of the Comprehensive Mobility Discharge Assessment Framework (CMDAF) when older adults are discharged from hospital-to-home.

**Methods:**

This protocol paper is an international modified e-Delphi study following the Recommendations for the Conducting and Reporting of Delphi Studies. International researchers, clinicians, older adults and family caregivers residing in a country with universal or near-universal health coverage will be invited to participate as ‘experts’ in three e-Delphi rounds administered through *DelphiManager©*. The e-Delphi Round 1 questionnaire will be developed based on scoping review findings and will be pilot tested. For each round, experts will be asked to rate factors for each determinant that are critical to assess as part of the CMDAF using a 9-point scale: Not Important (1–3), Important but Not Critical (4–6), and Critical (7–9). The scale will include a selection option of "unable to score" and experts will also be asked to provide a rationale for their scoring and suggest missing factors. Experts will receive feedback summaries in Rounds 2 and 3 to guide them in reflecting on their initial responses and re-rating of factors that have not reached consensus. The criteria for reaching consensus will be if ≥70% of experts rate a factor as "critical" (scores ≥7) and ≤ 15% of experts rate a factor as "not important" (scores≤ 3). Quantitative data will be analyzed using median values, frequencies, percentages, interquartile range, and bar graphs; Wilcoxon matched-pairs signed-rank test will be used to assess the stability of participants’ responses. Rationale (qualitative data) provided in the open-ended comments section will be analyzed using content analysis.

**Conclusion:**

This study is a first step in developing the CMDAF and will be used to guide a subsequent e-Delphi survey to decide on the tools that should be used to measure the examples of each factor included in our framework.

## Introduction

Globally, about 250 million older adults (defined as ≥65 years) have at least one disability [1], with about one-third of older adults reporting a mobility-related disability such as difficulty walking 400-meters or climbing a flight of stairs without assistance [2–4]. The worldwide prevalence of mobility-related disability among older adults living in high-income countries ranged from 22.6 to 47.6% between 2005–2015 [5–8]. According to the 2012 Canadian Survey on Disability, 20.6% of Canadians ≥65 years and 27% of Canadians ≥75 years have disabilities related to mobility [9]. Mobility-related disabilities often precede the onset of difficulties with activities of daily living and participation restrictions, often leading to social isolation, anxiety, and depression in older adults [2–4]. Mobility preservation among older Canadians is crucial in maintaining function and preventing further disability.

Mobility is defined as the ability to move between a variety of environments such as room, home, outdoors, neighborhood, community and the world either independently, with the use of assistive devices, or via transportation [10]. Historically, mobility has been viewed from a biomechanical or physiological perspective, which in turn has focused interventions on improving mobility-related physical outcomes such as gait, and muscle strength and power [11]. Over the years, the role of cognitive, financial, environmental, personal, psychological and social factors on mobility has been explored [10, 12, 13].

Webber and colleagues [10] in their Conical Model of Theoretical Framework for Mobility in older adults conceptualizes cognitive, psychosocial, physical, environmental, financial and personal histories/stories as determinants of mobility across seven life space locations—the room where the older adult sleeps, home, outdoors, neighborhood, service community and the surrounding area, and the world. Physical determinants of mobility include the number of chronic conditions, physical activity levels and muscle strength [12, 13]; while cognitive determinants include memory, executive functioning and mental status [12, 13]. Psychological determinants of mobility include depression, fear, anxiety, whereas social determinants include social networks and loneliness [12, 13]. Environmental factors are physical characteristics such as distance, temporal characteristics, light and weather conditions [13], and/or social, environmental policies such as public attitudes, social policies, services and systems, that hinder mobility [10]. Financial determinants of mobility may include personal income or household income, while personal determinants include age, gender, sex, marital status, race, ethnicity and culture [10].

Several studies have tested [12, 13] or expanded [14] the Conical Model of Theoretical Framework for Mobility in older adults. While Meyer et al. [12] and Giannouli [13] ’s studies highlighted the additive influence of cognitive, environmental, financial, personal, physical, psychological, and social factors on older adults’ mobility, Franke and colleagues reframed the Conical Model of Theoretical framework for Mobility into a sliding scale that reveals the dynamic, fluid and experiential nature of Mobility by analyzing physiological, subjective and contextual factors within and between people and their environment, over time [14]. Although Franke et al. [14] provided examples of how older adults and their caregivers can identify and rate each physiological, subjective, and contextual factor influencing their mobility across the sliding scale in a continuum scale of high to low, its application in a clinical setting is limited. For instance, this rating can be challenging for older adults with cognitive impairment. In addition, the reframed framework did not provide, for example, the specific physical factors, such as muscle strength, muscle power, range of motion, or built environment, such as street, residential and sideway characteristics, that the older adults or family members can identify as factors that influence their mobility.

Older adults experiencing a hospital-to-home transition are at increased risk for poor health outcomes such as mobility decline and deterioration in cognitive and functioning [15–17]. Between 30% to 60% of older adults experience functional decline after hospitalization [15–17], and mobility difficulties have been identified as predictors of mortality and loss of independence among community-dwelling older adults [18]. Functional deficits have been identified as independent risk factors for hospital readmission for older adults [19]. Therefore, efforts to improve mobility for older adults transitioning from hospital-to-home in the community are important and may help reduce hospital readmission costs. For instance, in Canada, the total cost of hospital readmissions is considerable at $1.8 billion per annum [20]. Older adults (65+ years) account for the largest proportion (60%) of total costs of hospital readmission in Canada [21], resulting in a significant economic burden to the Canadian health care system. Older adults readmitted to the hospital are at risk for hospital-acquired infections, deconditioning, and poor quality of life [20, 22]. Additionally, family caregivers are negatively affected and experience a sense of powerlessness, often resulting in anxiety and depression, when an older adult is readmitted to the hospital [23].

To date, hospital-to-home transition models such as Naylor [24] and Coleman [25] have typically focused on interventions to: improve provider-to-provider or provider-to-patient/family caregiver communication; improve coordination of care; and educate patients to self-monitor and manage their medical conditions. However, there has been little or no emphasis on addressing mobility [26], even though readmission risk prediction models targeting functional status consistently out-perform models based on medical comorbidities [27, 28]. Currently, mobility is rarely assessed during hospital discharge. Polnaszek et al. [29] reported that mobility-related recommendations, including assessment, were omitted in 53% of discharge summaries of 163 high-risk patients when transferred from hospital to sub-acute care facilities. In addition, among 64 mobility measures included in a review, none incorporated or highlighted the role of all seven mobility determinants [30], even though the interrelationship of these seven mobility determinants explains the complexity associated with mobility and could highlight which factors clinicians should intervene when older adults are discharge from the hospital. Addressing older adults’ mobility during hospital-to-home transitions is important because there is increasing recognition that a standardized, comprehensive functional assessment tool is necessary to improve the complex discharge process during the hospital-to-home transition [26, 31]. The World Health Organization’s Integrated Care for Older People Report recommended that every older person undergo continuous comprehensive assessments at services or system levels, including hospital discharge, to optimize their functional ability [26, 31].

To advance the use of the Conical Model of Theoretical Framework for Mobility [10], there is a need to develop specific factors for each determinant that are critical to assess when older adults are being discharged from hospital-to-home. This research will close this knowledge gap by developing a **C**omprehensive **M**obility **D**ischarge **A**ssessment **F**ramework (CMDAF) that clinicians can use during the hospital-to-home transition. The CMDAF will consist of factors within each mobility determinant and their corresponding outcome measures. The first phase of creating the CMDAF is to determine through consensus which factors within each determinant are critical to be assessed. The second phase is to identify the outcome measures for each of the factors within each determinant that reached consensus in the first phase. This protocol focuses only on the first phase. This e-Delphi study aims to prioritize and reach consensus on the factors for each mobility determinant [cognitive, financial, environmental, personal, physical, psychological, social] that are critical to assess as part of the CMDAF when older adults are discharged from hospital-to-home.

## Methods

### Study design

We will use modified Delphi methods to develop the CMDAF [32], following the Recommendations for the Conducting and Reporting of Delphi Studies (CREDES) to conduct the study and report our findings [33]. The Delphi method is a systematic approach to combining opinions to achieve consensus among individuals with a range of knowledge, experience and expertise through iterative, multi-stage completion of survey questionnaires (referred to as rounds) [32, 34]. Delphi methods are appropriate for this study because of the complexity of mobility, the need for group involvement and inclusion of a diversified and broad representation of expert opinions, including older adults and caregivers, to achieve the study aim [32].

We will use an e-Delphi method, online web-based survey questionnaires, for consensus building [32]. Participants worldwide (henceforth referred to as experts or expert participants) will be anonymous to each other to ensure one or more individuals do not dominate the process [32]. They will effectively connect with other experts without the need to be physically present, to identify, clarify, and refine their opinions and apply their expertise regarding older adult mobility during the hospital-to-home transition to reach consensus feasibly and cost-effectively [35].

### Sample–expert description and eligibility

Experts will include researchers, clinicians, older adults and family caregivers. Within the Delphi process, the definition of "experts" is fundamental to ensure the reliability of the Delphi findings. However, the definition of “experts" is highly debated in the Delphi research [36]. Baker and colleagues provided elements for defining "experts" for use within Delphi panel research, including specific qualifications, years of experience, and number of publications in the area of expertise [36]. We define each expert in our study following these elements. Researchers will be considered an "expert" if they have authored at least two peer-reviewed articles as either the first or senior author in at least one of the mobility determinants. Clinicians will be considered an "expert" if they have at least two years of clinical experience working with older adults with mobility difficulties in their field of professional expertise and hospital-to-home transitions. Older adults (65 years and older) will be considered an "expert" with lived experience if they self-identify as having at least one year of mobility difficulties and have experienced a hospital-to-home transition. Family caregivers will be considered an "expert" if they have at least one year experience providing informal care for older adults with mobility limitations, specifically during hospital-to-home transitions. This heterogeneous sample is needed to ensure that a spectrum of opinions from different stakeholders is included [32].

Experts are eligible to participate if they: a) conform eligibility based on the description above; b) can read and write in the English language; c) have knowledge of and ability to use computers and have reliable internet access; d) indicate interest, willingness and availability for participation in the timeframe of the three rounds of e-Delphi [34, 37]; e) reside, practice or research in a country with universal (or near-universal) health coverage such as Australia, Canada, United Kingdom [38]. Universal (near-universal) health coverage countries offer all their citizens affordable access to a comprehensive health service package [39]. This inclusion criterion ensures we recruit experts from countries with a healthcare system similar to Canada.

#### Sampling strategies and recruitment

The Delphi method employs criterion-based purposive and snowball sampling techniques to recruit experts [34]. Experts will be recruited from an international community of researchers, health and social care professionals, older adults and family caregivers based on the criteria described above.

We will use the following strategies for recruiting experts. First, we will send email invitations to the first and senior authors of the included articles in our scoping reviews. We will also employ snowball recruitment techniques by encouraging the pool of potential experts to send us the name and email contact of other potential participants who meet our expert description/criteria [32]. We will leverage the extensive networks of our Steering Committee members (described below) [40], with each Steering Committee member providing a list of additional potential experts that will receive an invitation to participate in the study. As needed, clinician and research experts will also be recruited through interdisciplinary professional associations such as Australian Association of Gerontology, British Society of Gerontology, Canadian Association of Gerontology, International Association of Gerontology and Geriatrics. Older adults will be recruited through HelpAge International [41], which comprises a pool of older adults residing in the countries with universal (near-universal) healthcare systems; and family caregivers will be recruited through family caregivers’ organizations such as IMAGINE Citizen Collaborating for Health [42], Caregivers Alberta [43]. We will approach these organizations to explain our study and aims and request they nominate potential experts to be invited to participate in our study. To ensure the required sample representation of experts, recruitment will be monitored, and all interested individuals will conform eligibility prior to participating.

#### Sample size

There is no set standard or accepted guidelines for the sample size for e-Delphi [32]; instead decision-making is guided by the scope and aims of the study and practicality [32]. A 2011 systematic review found that the median number of individuals invited to participate in Delphi studies was 17, with a range of 3 to 418 (Q1 = 11, Q3 = 31) [44]. Based on this, we will aim to include at least 20 experts (five per stakeholder group—researchers, clinicians, older adults and family members).

Data regarding specific recruitment efforts such as number of invitations sent for Delphi and e-Delphi studies and response rates for these efforts are sparse in the literature [33, 45], in particular for specific stakeholder groups. Often, recruitment response rate is calculated as the number of participants who complete the Delphi rounds (participation rates), and these rates and reporting of rates vary greatly. For instance, recruitment response ranged 46% to 65% for participant groups not specified [46]; 96% for patients and caregivers and 81% for research experts [47]; and 100% for all participant groups [48]. Baldwin et al. [49] reported an overall response rate of 69.5% to expression of interest invitations across a broad range of experts, including patients, while Stewart et al. [50] reported a response rate of 67.9%. Using recruitment response rates of 65%, we will approach approximately 31 experts to achieve our sample size of 20, ensuring the equal number of experts across the four stakeholder groups: clinicians, researchers, older adults and family caregivers.

### Steering committee

The Steering Committee, including team investigators, an older adult and a family caregiver, will provide overall study oversight. The Committee comprises members from various disciplines such as nursing, physiotherapy, gerontology, with clinical and research expertise related to older adults, mobility, hospital-to-home transitions, and qualitative, quantitative, and Delphi methods. The older adult will have lived experience with mobility limitation and transitioning from hospital-to-home, while the family caregiver will have lived experience providing care to an older adult with mobility limitation and hospital-to-home transition. The Steering Committee will meet at key stages throughout the study and will be responsible for identifying and responding to any issues arising, reviewing study conduct, and overseeing knowledge dissemination. The Steering Committee will conduct content and face validity of e-Delphi questionnaire to ensure accuracy, comprehensiveness, clarity of wording, and appropriateness of structure; review results at each round; and review feedback summaries to be provided to experts at subsequent rounds. The Steering Committee may also be required to decide on items should experts fail to reach consensus [40]. Any decisions made by the Steering Committee will be communicated to the expert participants throughout the study, and experts will be provided with opportunities to respond. This approach reduces the burden on the experts during the Delphi process [34].

### General procedure

**Fig 1:**
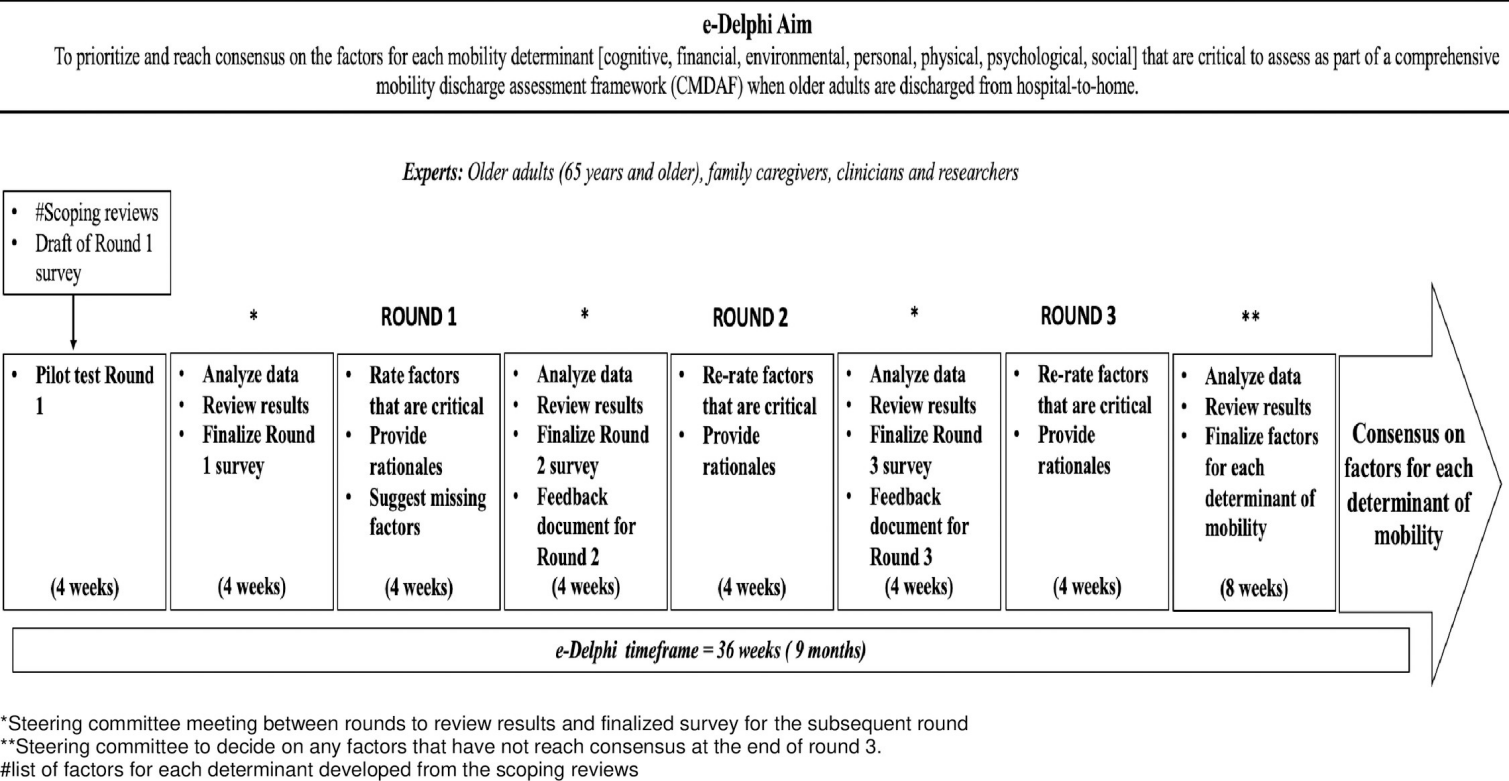
e-Delphi survey process and timeline.

While the classical Delphi typically uses four rounds [34], two or three rounds have been used to achieve a consensus [33, 51]. Because of the topic’s complexity, our experts’ heterogeneity, and our aims, experts might not achieve consensus in two rounds. We anticipate that at least three rounds will be needed to achieve consensus, as prolonged Delphi process, for example four rounds, often leads to reduced response rate, which affects the Delphi process’s validity (Fig 1).

The survey questionnaire for each e-Delphi round will include: background information containing study’s purpose, aims, description of e-Delphi methods, and definitions of factors within each determinants; questions to collect demographic and participant information; and, the e-Delphi questionnaire consists of structured and open-ended questions. We will use a 9-point scale, divided into three categories, for importance rating: Not Important (1–3), Important but Not Critical (4–6), and Critical (7–9) [52]. As well, an “Unable to Score” response and instruction for its use will be provided should experts feel uncomfortable rating any particular question. A 9-point scale is preferred in a Delphi survey as it increases sensitivity and consensus can be achieved on more items compared to 3- or 5-point scale [53].

The survey will be administered through *DelphiManager* [54], a web-based system designed to facilitate the building of e-Delphi surveys that includes functionality that allows for easy and efficient data management. *DelphiManager* allows for secure data collection and integrity using multiple encryption layers, and "quasi anonymity". Quasi-anonymity refers to researchers knowing the experts’ identity and their responses, but experts are anonymous to each other and provide responses that are anonymous to each other [55]. For each round, experts will receive a link to an e-Delphi questionnaire in *DelphiManager*. Only experts who provide informed consent will have access to the questionnaire.

All experts, including older adults and family caregivers, will be asked to complete questions regarding each mobility determinant. As experts, their opinions and perspectives across all determinant domains lend trustworthiness and relevance to our study [37]. Research has demonstrated that older adults and their family caregivers can actively participate in an e-Delphi consensus, especially when plain language explanations of items for consensus are provided [56]. A description of each factor will be provided in each round of the e-Delphi.

Although there is no specific recommended duration for each round of an e-Delphi process [35], Junger et al. [33] noted that Delphi round durations could range from 10 days to 10 weeks. Based on previous international e-Delphi studies with multi-stakeholders [35, 40, 57], we estimate that each round will be open for four weeks to ensure experts have enough time to participate effectively, without losing interest [35]. There will be a minimum four-week time interval between rounds to allow for data analysis, review of results by the Steering Committee, revision of the questionnaire and development of the feedback document for subsequent rounds (see Fig 1). The feedback document will include a summary document of preceding rounds, including bar graphs showing experts’ group ratings, distribution of ratings, and individual ratings on factors that reached consensus and did not reach consensus and a qualitative summary of the rationale for each rating. Providing a feedback document to experts for subsequent rounds is an important component of the process because it allows experts to consider the group rating compared to their response enabling them to reflect, revise or change their opinions while avoiding the effects of dominant individuals that could influence their decision in direct communication (e.g. face-to-face communication) [32, 37]. We anticipate that the e-Delphi process will occur over approximately nine months, including the test round (see Fig 1).

#### Test round (pilot study)

Prior to Round 1 of the e-Delphi, the survey questionnaire will be pilot tested (test round) to: ensure content and face validity [58]; receive feedback regarding format, comprehensiveness, clarity of instructions, descriptions/definitions and participation time [34]; and, test the online platform and its nuances [37]. We will recruit four experts (a clinician, a researcher, an older adult and a family caregiver) to pilot test the questionnaire. Experts involved in the test rounds will not participate in subsequent rounds.

#### e-Delphi round response rate

No agreed-upon guidelines exist for an acceptable response rate for each round of the e-Delphi process [32]. A 70% response rate has been suggested in the literature to maintain rigour in a Delphi technique [32].

#### Strategies to increase the response rate

We will incorporate Dillman’s Tailored Design Method [59] suggestions and published recommendations [35, 45, 59], to encourage participation and engagement with each round, and to ensure the high retention of expert participants in the e-Delphi process to achieve our minimum 70% response rate [32]. Strategies we will employ to increase response rates and thus decrease potential attrition bias include: sending an initial personalized email to the potential experts requesting participation before sending the e-Delphi survey [35, 59]; emphasizing that each expert’s perspective matters, and for the result to be meaningful, completing the whole e-Delphi process is important [59]; and sending thank you emails to experts who have participated [45]. For each round, we will send weekly personalized reminders for the first three weeks following the distribution of the survey [59].

#### Consensus level

Although there is no universally accepted threshold for defining consensus in an e-Delphi process, establishing an a priori consensus criteria is considered an indicator of a good quality Delphi process [33, 60]. Researchers have established consensus using a formal measure of agreement, a measure of central tendency, percentage agreement, a central tendency within a specific range (restricted or unrestricted), the proportion of range (restricted or unrestricted), decrease in variance, stability and rank [51]. While there is no agreement on the best approach, the percentage agreement and the proportion within a range are most often used in Delphi studies [51]. In this study, we define consensus as ≥70% of experts rated a factor as “critical” (scores ≥7) and ≤15% of experts rated a factor as “not important” (scores≤ 3).

#### Questions regarding the e-Delphi process or rounds

Experts will be informed that they are welcome to contact the research team at any time with questions including clarification about the aims, instructions, e-Delphi process, descriptions or definitions, and technical support when completing the survey.

#### e-Delphi rounds

Evidence from scoping reviews will inform Round 1 of our e-Delphi study rather than using general, open-ended questions for idea generation as is typical of classical Delphi studies [32, 61]. Participants will be invited to rate 76 factors: cognitive (n = 5), environmental (n = 17), financial (n = 3), personal (n = 11), physical (n = 20), psychological (n = 15), and social factor (n = 5) identified through the scoping reviews. Experts will receive compiled lists and descriptions of factors for each determinant identified from the scoping reviews.

#### Round 1

e-Delphi Round 1 questionnaire will be developed based on the findings of the scoping reviews. Experts will be asked to: a) rate factors for each determinant that are critical to assess as a part of a CMDAF when older adults are discharged from hospital to home using a 9-point GRADE Scale of Not Important (1–3), Important but Not Critical (4–6), and Critical (7–9); with the option of selecting “unable to score” b) provide a rationale for their ratings in an open-ended comment section; and, c) suggest missing factors for each mobility determinant.

#### Round 2

We will provide a feedback summary based on the Round 1 responses, and the feedback summary will be included as part of Round 2’s introductory message. The feedback document will contain a summary document of Round 1 including bar graphs showing experts’ group ratings, distribution of ratings, and individual ratings on factors that reached consensus and did not reach consensus [48], suggested factors from Round 1 and any possible changes on factor descriptions based on experts’ comments. In Round 2, experts will be asked to rate the factors suggested by experts in Round 1 and re-rate the factors that did not reach consensus while considering the feedback [32, 47, 50].

#### Round 3

Experts will receive a similar feedback document based on findings from Round 2. Experts will be asked to re-rate only items that did not reach consensus by reflecting on, verifying, or modifying their original choice, and provide rationale for their choices.

After Round 3, the Steering Committee group will review the results and make any decisions as necessary (e.g., whether to include or exclude any factor(s) that has not reached a consensus, but is close to reaching consensus) [40]. Any decision-making by the Steering Committee will be communicated back to the experts [34]. A final list of factors for each mobility determinant will be collated to form a part of the CMDAF.

### Data analysis

While expert participants will be able to withdraw at any time, data will be included up to the point of round withdrawal. All quantitative analysis will be conducted using STATA,v16.1© [62]. Descriptive statistics will be used to analyze demographic data and quantitative items. Continuous data will be tested for normality before the use of parametric inferential statistics. The response rate for each round will be calculated by dividing the number of usable responses returned by the total number of invitations sent out multiplied by 100% at each round [57, 63]. Median values, frequencies and percentages will be used as indicators of agreement on the 9-point scale [32], and will be used to calculate consensus level. The interquartile range and bar graphs [49] will show the dispersion levels and individual ratings at each round for each factor [32]; this will enable experts to see where their responses stand in relation to the group’s responses. Stability, defined as the consistency of responses between successive rounds [61], will be used to assess the shift in scores across rounds as a consequence of considering the anonymized feedback from other expert participants, and will be calculated using Wilcoxon matched-pairs signed-rank or interclass correlation coefficient, if the data is normally distributed [61]. The analysis will be completed for all experts as a group and separately for each expert [49].

Qualitative analyses will be managed using NVivo Software© [64]. Open-ended comments such as rationale for experts’ choices or changes, will be analyzed using content analysis [65]. Two coders will read the responses independently to develop codes and themes for each round. Coders will meet to merge their themes. Any disagreement will be discussed during a Steering Committee meeting and resolved. A reflexive journal and audit trail will be kept to capture methodological decisions throughout the Delphi process [32].

### Ethical considerations

This Research have been approved by Hamilton Integrated Research Ethics Board (HIREB Project no: 7212) on May-06-2021. Only invited experts who provide informed consent will be eligible to participate in the study. The privacy and identity of all experts will be protected during and after the study. We will de-identify any feedback or summary statements shared with the experts.

## Discussion

Mobility deficits are often missed during the hospital-to-home transition for older adults [66]. Functional status is a better predictor of hospital readmission than medical comorbidities in a medically complex rehabilitation population [27, 28]. Empirical evidence has found that mobility-related recommendations were omitted entirely in 53% and partially omitted in 47% of discharge summaries of 163 high-risk patients transitioning from hospital to subacute care facilities [29]. No comprehensive assessment framework based on the Conical Model of Theoretical Framework for Mobility currently exists. Similarly, available assessment tools do not incorporate all seven determinants. For instance, the Activity Measure for Post-Acute Care Inpatient Mobility Short Form [67] was designed to assess three activity domains of post-acute care function: movement and physical such as body position and transfers, personal and instrumental such as personal care, home skills, and applied cognition such as speaking and understanding; but does not include domains related to social, psychological, financial, and environmental factors affecting mobility. With no comprehensive mobility discharge assessment framework consisting of the seven determinants of mobility, it is challenging for healthcare workers to implement an integrated, holistic approach to examination, decision-making and recommendations incorporating factors known to be associated with mobility decline in older adults after discharge. Therefore, the proposed CMDAF, which will be evidence and stakeholder informed, will address the complexity of mobility in older adults during the hospital-to-home transition. It will serve as a guide for an all-inclusive assessment of mobility-related issues at hospital discharge, thereby reducing the number of readmissions and their related economic and personal burden. This study is a first step in the development of the CMDAF and will be used to guide a subsequent e-Delphi survey to decide on the tools that should be used to measure the examples of each factor included in our framework.
